# Lifespan Changes of the Human Brain In Alzheimer’s Disease

**DOI:** 10.1038/s41598-019-39809-8

**Published:** 2019-03-08

**Authors:** Pierrick Coupé, José Vicente Manjón, Enrique Lanuza, Gwenaelle Catheline

**Affiliations:** 1 University Bordeaux, LaBRI, UMR 5800, PICTURA, F-33400 Talence, France; 2 CNRS, LaBRI, UMR 5800, PICTURA, F-33400 Talence, France; 3 Instituto Universitario de Tecnologías de la Información y Comunicaciones (ITACA), Universitat Politècnica de València, Camino de Vera s/n, 46022 Valencia, Spain; 4 University Valencia, Department of Cell Biology, Burjassot, 46100 Valencia, Spain; 5 University Bordeaux, CNRS, EPHE, PSL, INCIA, UMR 5283, F-33000 Bordeaux, France

## Abstract

Brain imaging studies have shown that slow and progressive cerebral atrophy characterized the development of Alzheimer’s Disease (AD). Despite a large number of studies dedicated to AD, key questions about the lifespan evolution of AD biomarkers remain open. When does the AD model diverge from the normal aging model? What is the lifespan trajectory of imaging biomarkers for AD? How do the trajectories of biomarkers in AD differ from normal aging? To answer these questions, we proposed an innovative way by inferring brain structure model across the entire lifespan using a massive number of MRI (N = 4329). We compared the normal model based on 2944 control subjects with the pathological model based on 3262 patients (AD + Mild cognitive Impaired subjects) older than 55 years and controls younger than 55 years. Our study provides evidences of early divergence of the AD models from the normal aging trajectory before 40 years for the hippocampus, followed by the lateral ventricles and the amygdala around 40 years. Moreover, our lifespan model reveals the evolution of these biomarkers and suggests close abnormality evolution for the hippocampus and the amygdala, whereas trajectory of ventricular enlargement appears to follow an inverted U-shape. Finally, our models indicate that medial temporal lobe atrophy and ventricular enlargement are two mid-life physiopathological events characterizing AD brain.

## Introduction

Alzheimer’s disease (AD) is the most prevalent form of dementia in persons older than 65 years^1^. Cognitive impairment, mainly related to memory deficits, is the most common manifestation of this disease^2^. Available neuroimaging evidence suggests that the neuropathological alterations underlying AD probably begin much earlier than the appearance of clinical symptoms and years before clinical diagnosis^3^. From these results, it appeared that the pharmacological management was finally implemented in patients with a largely advanced neurodegenerative process, making it difficult to fight against pathological progression. In this context, the concept of disease-modifying therapies is emerging and the search for early biomarkers of these alterations is currently a hot topic of research^4^.

Neurodegeneration, assessed by the level of cerebral atrophy, is one of these biomarkers. In recent decades, several MRI studies have investigated neurodegeneration in the prodromal phase of Alzheimer’s disease^5,6^. However, very few of them attempted to investigate the preclinical phase of the disease, the very early asymptomatic phase. Indeed, this type of study is a very challenging task since it requires an imaging database starting before the appearance of the disease, and the corresponding long follow-up study. Therefore, in the past, such studies have been based on subjects with rare autosomal dominant mutations associated with a high risk of developing dementia^7–10^ or on longitudinal studies with long follow-up in which brain imaging have been performed before the appearance of clinical symptoms (i.e., memory impairment)^11–13^. In these previous long follow-up studies, the starting point of the neurodegeneration was not determined since incident cases already present brain morphometric differences at baseline 7 or 10 years before the diagnosis^13–15^ or 5 before the apparition of Mild Cognitive Impairment (MCI)^16^. Finally, the lifespan evolution of these imaging biomarkers many years before cognitive decline also remains unknown.

Determining the timing of the onset of neurodegeneration would require longitudinal MRI datasets with several decades of follow-up. So far, due to obvious technical reasons, such imaging dataset does not exist yet. Consequently, in this paper, we propose to consider alternative questions such as – When does the AD model diverge from the normal aging model? How do the trajectories of biomarker models differ in AD from normal aging? To answer these questions, we present an innovative approach based on an extrapolated lifespan model of AD brain structures using large-scale databases. To this end, we propose to take advantage of the new paradigm of BigData sharing in neuroimaging^17^ by analyzing publically available databases including subjects from a wide age range covering the entire lifespan. Such analysis can be very valuable since epidemiological studies indicate that late dementia is associated with early exposure to risk factors at midlife, highlighting the need to consider brain biomarkers throughout the entire lifespan^18–20^.

Recently, we used BigData approach to propose an analysis of brain trajectory across the entire lifespan using N = 2944 MRI of cognitively normal subjects (CN)^21^. Herein, we present a study following a similar approach to analyze the lifespan changes of the human brain in AD. To this end, we propose to build an extrapolated model of AD for brain structures. We assume that the neurodegenerative process is slow and progressive. The slow accumulation of Amyloid-β^3,22,23^ and the smooth atrophy of brain^3,10^ seem to indicate that brain alterations occurs progressively in AD. Accordingly, to build our lifespan AD model we combined young CN with aged AD and MCI patients. We used 1385 MRI of AD and MCI patients (from 55 y to 96 y) and 1877 MRI of CN subjects younger than them (from 9 months to 55 y). In our approach, we used CN subjects as young asymptomatic AD subjects to overcome the absence of datasets including young CN who will develop AD several decades latter. However, MRI studies have showed brain alterations several years before diagnosis or MCI stage^14–16^. Therefore, the proposed approach can be viewed as a conservative lifespan model of AD. In this study, we first propose a comparison of lifespan evolution of global white and gray matter and subcortical structures between AD sample and CN sample. Afterward, we focus on temporal lobe structures such as the hippocampus and amygdala – known to be affected in AD^24,25^ and the lateral ventricles, also a known AD biomarker^26,27^ in a supplementary analysis considering three pathological models: an AD model composed of 2303 subjects, an MCI model composed of 2836 samples subjects and an AD/MCI model composed of 3262 subjects.

## Material and Methods

### Groups definition

This study aims at comparing normal and pathological models of brain structure across the entire lifespan. To this end, models were estimated on four different groups to generate CN, AD/MCI, AD and MCI trajectories.

- For the CN model, we used the N = 2944 subjects from 9 months to 94 y of the cognitively normal dataset as done in^21^.
- For the AD/MCI model, we used N = 3262 subjects. We mixed AD patients, with MCI patients and with young CN considered as presymptomatic subjects. We used 426 AD patients (from 55 y to 96 y), 959 MCI patients (from 55 y to 92 y) of the AD/MCI dataset and all the CN younger than 55 y (i.e., 1877 subjects). These subjects are included in the CN used for CN trajectory.
- For the AD model, we used N = 2303 samples. We mixed AD patients with young CN. More precisely, we used 426 AD patients (from 55 y to 96 y) of the AD/MCI datasets and all the CN younger than 55 y (i.e., 1877 subjects).
- For the MCI model, we used N = 2836 samples. Here, 959 MCI patients (from 55 y to 92 y) of the AD/MCI datasets were mixed with all the CN younger than 55 y (i.e., 1877 subjects).

### Datasets description

To study structural changes across the entire lifespan for CN and AD, we aggregated several open access databases to construct two datasets. In the following, CN and AD/MCI datasets will be described. The four previously described groups are built on these datasets.

#### Cognitively normal dataset (N = 2944)

The cognitively normal dataset is composed of the 3296 T1-weighted (T1w) MRI. The composition of the nine open access databases used to build the control dataset is provided in Table 1. As explained in^21^, after a quality control (see image processing section), only 2944 MRI were kept. The female proportion is 47% for the remaining subjects and the age range is [0.75–94] years.

**Table 1:** Dataset description for CN data.

Dataset	Before QC	After QC	Gender after QC	Age in years after QC
C-MIND	266	236	F = 129 M = 107	8.44 (4.35) [0.74–18.86]
NDAR	612	382	F = 174 M = 208	12.39 (5.94) [1.08–49.92]
ABIDE	528	492	F = 84 M = 408	17.53 (7.83) [6.50–52.20]
ICBM	308	294	F = 142 M = 152	33.75 (14.32) [18–80]
IXI	588	573	F = 321 M = 252	49.52 (16.70) [20.0–86.2]
OASIS	315	298	F = 187 M = 111	45.34 (23.82) [18–94]
AIBL	236	233	F = 121 M = 112	72.24 (6.73) [60–89]
ADNI 1	228	223	F = 108 M = 115	75.96 (5.03) [60–90]
ADNI 2	215	213	F = 113 M = 100	74.16 (6.39) [56.3–89]
Total	**3296**	**2944**	**F** = **1379 (47%)** **M** = **1565 (53%)**	**39.65 (26.62)** **[0.74–94]**

This table provides the name of the dataset, the MR acquisition configuration, the number of considered image before and after QC, the gender proportion after QC and the average mean, standard deviation in parentheses and the interval in brackets.

#### AD/MCI dataset (N = 1385)

The AD/MCI dataset is composed of 426 AD patients and 959 MCI patients extracted from OASIS, AIBL, ADNI1 and ADNI2 databases. Details on clinical criterion for groups definition are provided in^28^ for ADNI1, ADNI2 and AIBL and in^29^ for OASIS. After a quality control, only 1385 MRI were kept. The female proportion is 44% for the remaining subjects and the age range is [55–96] years (see Table 2).

**Table 2:** Dataset description of AD and MCI data.

Dataset	Before QC	After QC	AD stage (MCI/AD) after QC	Gender after QC	Age in years after QC
OASIS	98	95	50/45	F = 56 M = 39	76.58 (7.18) [62–96]
AIBL	112	106	59/47	F = 58 M = 48	74.15 (7.80) [55–93]
ADNI 1	587	568	385/183	F = 225 M = 343	75.04 (7.41) [55–91]
ADNI 2	621	616	465/151	F = 270 M = 346	72.56 (7.64) [55–90]
Total	**1418**	**1385**	**959/426**	**F** = **609 (44%)** **M** = **776 (56%)**	**73.7 (7.84)** **[55–96]**

This table provides the name of the dataset, the MR acquisition configuration, the number of considered image before and after QC, the gender proportion after QC and the average mean, standard deviation in parentheses and the interval in brackets.

In the following, more details are provided about acquisition protocols of the different datasets used in this study.

- **C-MIND:** 266 images of control subjects from the C-MIND dataset (https://research.cchmc.org/c-mind/) are used in this study. All the 3D T1-weight (T1w) MPRAGE high-resolution MRI were acquired at the same site on a 3 T scanner with spatial resolution of 1 mm^3^ acquired using a 32 channel SENSE head-coil.
- **NDAR:** 415 of control subjects from the Database for Autism Research (NDAR) (https://ndar.nih.gov) are used in this study. The T1w 3D MRI were acquired on 1.5 T MRI and 3 T scanners. In our experiments, we used the NIHPD (http://www.bic.mni.mcgill.ca/nihpd/info/data_access.html) dataset and 197 images of control subjects from the Lab Study 19 of National Database for Autism Research. For the NIHPD dataset, the 3D T1w SPGR MRI were acquired at six different sites with 1.5 Tesla systems by General Electric (GE) and Siemens Medical Systems with spatial resolution of 1 mm^3^. The 3D T1w MPRAGE MRI from the Lab Study 19 were scanned using a 3 T Siemens Tim Trio scanner at each site with spatial resolution of 1 mm^3^
- **ABIDE:** 528 control subjects from the Autism Brain Imaging Data Exchange (ABIDE) dataset (http://fcon_1000.projects.nitrc.org/indi/abide/) are used in this study. The MRI are T1w MPRAGE acquired at 20 different sites on 3 T image and the details of acquisition, informed consent, and site-specific protocols are available on the website.
- **ICBM**: 308 normal subjects from the International Consortium for Brain Mapping (ICBM) dataset (http://www.loni.usc.edu/ICBM/) obtained through the LONI website are used in this study. The T1w MPRAGE MRI were acquired on a 1.5 T Philips GyroScan imaging system (Philips Medical Systems, Best, The Netherlands) with spatial resolution of 1 mm^3^.
- **IXI**: 588 normal control from Information eXtraction from Images (IXI) database (http://brain-development.org/ixi-dataset/) are used in this study. The MRI are T1w images collected at 3 sites with 1.5 and 3 T scanners with spatial resolution close to 1 mm^3^.
- **OASIS:** 315 control subjects and 98 AD/MCI patients from the Open Access Series of Imaging Studies (OASIS) database (http://www.oasis-brains.org) are used in this study. The MRI are T1w MPRAGE image acquired on a 1.5 T Vision scanner (Siemens, Erlangen, Germany) and resliced at 1 mm^3^.
- **ADNI1:** 228 control subjects and 587 AD/MCI patients from the Alzheimer’s Disease Neuroimaging Initiative (ADNI) database (http://adni.loni.usc.edu) phase 1 are used in this study. These baseline MRI are T1w MPRAGE acquired on 1.5 T scanners at 60 different sites across the United States and Canada with reconstructed spatial resolution of 1 mm^3^.
- **ADNI2:** 215 control subjects and 621 AD/MCI patients from the ADNI2 database (second phase of the ADNI project) are used in this study. The baseline MRI are T1w MPRAGE acquired on 3 T MR scanners with the standardized ADNI-2 protocol (www.loni.usc.edu) with spatial resolution close to 1 mm^3^.
- **AIBL**: 236 control subjects and 112 AD/MCI patients from the Australian Imaging, Biomarkers and Lifestyle (AIBL) database (http://www.aibl.csiro.au/) are used in this study. The baseline MRI are T1w image acquired on 3 T MR scanners with the ADNI protocol (http://adni.loni.ucla.edu/research/protocols/mri-protocols) and with custom MPRAGE sequence on the 1.5 T scanners.

### Image processing

All the considered images were processed with the volBrain pipeline^30^ (http://volbrain.upv.es). The volBrain system is a web-based online tool providing automatic brain segmentation and generating report summarizing the volumetric results. The full processing time is around 10 minutes. In the past 2 years, volBrain has processed online more than 75.000 brains for approximately 1800 users. In a recent work, we compared volBrain pipeline with two well-known tools used on MR brain analysis (FSL and Freesurfer). We showed significant improvements in terms of both accuracy and reproducibility for intra and inter-scanner scan-rescan acquisitions^30^. The volBrain processing pipeline includes several steps to improve the quality of the input MR images and to homogenize their contrast and intensity range^30^. The volBrain pipeline achieves the following preprocessing steps: (1) denoising using spatially adaptive non-local means^31^, (2) rough inhomogeneity correction using N4 method^32^, (3) affine registration to MNI152 space using ANTS software^33^, (4) SPM based fine inhomogeneity correction^34^ and (5) tissue based intensity standardization^35^. After preprocessing, the brain is segmented into several structures at different scales. First, the total intracranial volume (TIV) is obtained with NICE method^36^. Then, tissue classification is performed using the TMS method^35^ and finally subcortical structures are estimated using the non-local label fusion method^37^. All the segmentation methods of volBrain are based on a library of 50 experts manually labelled cases (covering almost the entire lifespan). It is worth to note that the used manual hippocampus labeling followed the EADC-ADNI harmonized protocol which is the current consensus protocol for hippocampus segmentation in AD^38^. More details about volBrain pipeline can be found in^30^. Finally, a multi-stage quality control (QC) procedure was performed to carefully select subjects included. First, a visual assessment was done for all input images by checking screen shots of one sagittal, one coronal and one axial slice in middle of the 3D volume. Then, a visual assessment of processing quality was carried out by using the volBrain report which provides screenshots for each step of the pipeline. Finally, a last control was performed by individually checking with a 3D viewer all outliers detected using the estimated model (see^21^ for more details).

### Statistical Analysis

Different model types were considered to estimate the final model of each structure. The candidate models were tested from the simplest to the most complex. A model type was kept as a potential candidate only when simultaneously F-statistic based on ANOVA (i.e., model vs. constant model) was significant (p < 0.05) and when all its coefficients were significant using t-statistic (p < 0.05). As in^21^, the following model types were used as potential candidates:

1. Linear model
  $$Vol={\beta }_{0}+{\beta }_{1}Age+\varepsilon $$
2. Quadratic model
  $$Vol={\beta }_{0}+{\beta }_{1}Age+{\beta }_{2}Ag{e}^{2}+\varepsilon $$
3. Cubic model
  $$Vol={\beta }_{0}+{\beta }_{1}Age+{\beta }_{2}Ag{e}^{2}+{\beta }_{3}Ag{e}^{3}+\varepsilon $$
4. Linear hybrid model: exponential cumulative distribution for growth with linear model for aging
  $$Vol={\beta }_{4}.(1-{e}^{-Age/{\beta }_{5}})+{\beta }_{0}+{\beta }_{1}Age+\varepsilon $$
5. Quadratic hybrid model: exponential cumulative distribution for growth with quadratic model for aging
  $$Vol={\beta }_{4}.(1-{e}^{-Age/{\beta }_{5}})+{\beta }_{0}+{\beta }_{1}Age+{\beta }_{2}Ag{e}^{2}+\varepsilon $$
6. Cubic hybrid model: exponential cumulative distribution for growth with cubic model for aging

$$Vol={\beta }_{4}.(1-{e}^{-Age/{\beta }_{5}})+{\beta }_{0}+{\beta }_{1}Age+{\beta }_{2}Ag{e}^{2}+{\beta }_{3}Ag{e}^{3}+\varepsilon $$

To select the best model type, we used the Bayesian Information Criterion among kept candidate models – p < 0.05 for ANOVA of the model vs. constant model and p < 0.05 for T-test of all the coefficients. The Bayesian information criterion is a measure providing a trade-off between bias and variance to select the model explaining most of the data with a minimum number of parameters. Moreover, to compensate for variability introduced by head size difference, models were estimated on normalized volume in % of total intracranial volume. Left and right volumes were added to obtain the final volume structure. The prediction bounds were estimated with a confidence level at 95%. This model selection procedure was applied to all the considered structures. In this study, we studied the following brain structures: lateral ventricles, hippocampus, amygdala, caudate, putamen, accumbens, globus pallidus and thalamus. Moreover, tissue classification was used to obtain the global volume of white matter and gray matter. All statistical tests were performed with Matlab© using default parameters. Afterwards, percentage of relative rate of change per year and percentage of abnormality were computed on the estimated models. The relative rate of change in percentage per year was computed as the first derivative of the model divided by the model^39^ and the abnormality in percentage as the absolute difference between pathological models and control model divided by control model (i.e., absolute relative difference compared to control).

Finally, we studied lifespan classification accuracy of several AD biomarkers. To this end, for each age, a classification was performed with a linear discriminate analysis (LDA) using all the samples in an interval of 10 y (i.e.,+/− 5 years). We used Area Under the Curve (AUC) as classification performance metric. The AUC was estimated through a cross-validation procedure based on a repeated K-fold using 10 iterations and 10-fold. Finally, the average AUC obtained over the 10 repetitions is reported for all ages. Classification experiments were performed with Matlab© using default parameters.

## Results

**Figure 1:**
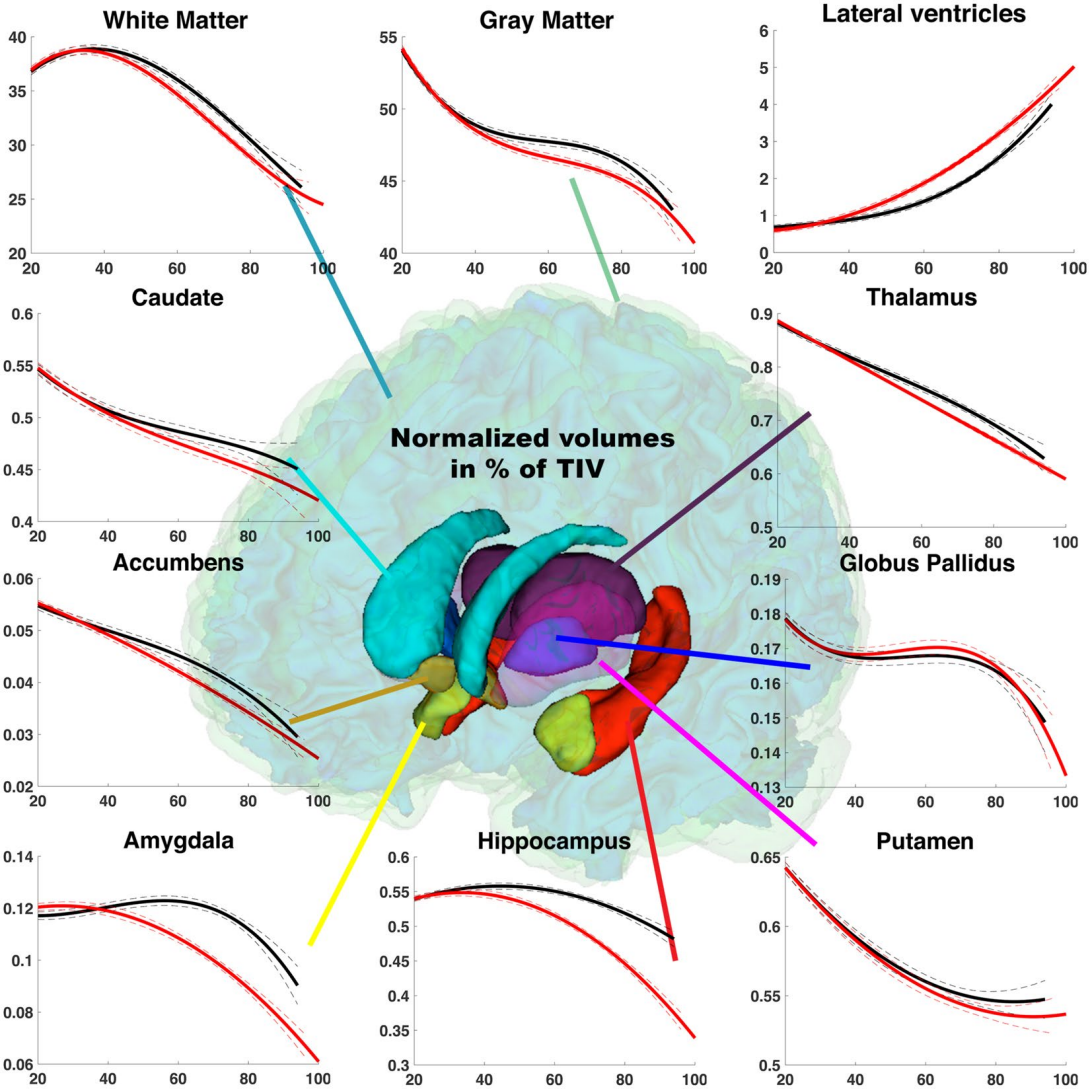
Models based on relative volumes (% total intracranial volume) for brain cortical and subcortical structures across the entire lifespan. These models are estimated according to the age of subjects. Model for CN group (N = 2944) is in black and model for AD/MCI group (N = 3262) is in red. The prediction bounds of the models are estimated with a confidence level at 95%.

Figure 1 presents models of all considered structures for AD/MCI and CN groups. This figure shows that hippocampus and amygdala models present marked divergences between AD/MCI and CN, and also indicates that this divergence increases with age. Moreover, the divergence of control and pathological models for these structures occurs early around 40–45 y. Lateral ventricles also exhibit early divergence – starting around 42 y – between both models, however the distance between models decreases at advanced ages. Similarly, the thalamus presents an early but weak divergence that decreases at advanced ages. Pathological models of caudate and accumbens nuclei exhibit accelerated volume decreases from 50–60 y. However, confidence intervals for these structures overlap again after 85 y (see Table 3). For white matter and gray matter, AD/MCI models present an early accelerated aging compared to CN models around 45 y. However, after 80 y, CN models of brain tissues show an accelerated volume decreases. Consequently, confidence intervals of pathological and normal models overlap after 85 years (see Table 3). Finally, normal and pathological models for globus pallidus and putamen present similar trends.

**Table 3:** Age range in years where confidence intervals of the predicted pathological models do not overlap with the predicted control models.

	CN vs. AD/MCI	CN vs. AD	CN vs. MCI
White Matter	[47.6–85.8]	[46.9–89.9]	[53.7–82.3]
Gray Matter	[45.0–85.6]	[46.2–86.4]	[58.3–86.7]
Lateral Ventricles	[42.0–93.2]	>**38.6**	[45.1–89.2]
Caudate	[62.7–84.1]	[68.8–82.8]	[70.3–84.7]
Putamen	N/A	N/A	N/A
Thalamus	[42.8–89.1]	[41.7–89.6]	[45.5–86.7]
Globus Pallidus	N/A	N/A	N/A
Hippocampus	> **39.0**	> **37.1**	> **42.4**
Amygdala	>**43.8**	>**40.2**	>**49.3**
Accumbens	[48.1–85.6]	[46.0–88.0]	[52.6–82.3]

The prediction bounds are estimated with a confidence level at 95%. Three model comparisons are presented CN (N = 2944) vs. AD/MCI (N = 3262), CN (N = 2944) vs. AD (N = 2303) and CN (N = 2944) vs. MCI (N = 2836). N/A for Non Applicable.

Table 3 shows the age ranges where the confidence interval of the predicted pathological models (i.e., AD, MCI and AD/MCI) do not overlap with the confidence interval of the control models.

First, only hippocampus and amygdala models present non-overlapping confidence intervals after divergence for all the studied pathological modes (i.e., AD/MCI, AD and MCI) (see Table 3). This is also valid for lateral ventricles model but only when using the AD group. For all other considered structures, predicted confidence intervals overlap again at advanced ages around 80–90 y.

Second, hippocampus is the deep gray structure showing the earliest model divergence at 39 y for AD/MCI, 37 y for AD and 42 y for MCI. Then, the lateral ventricles models exhibit a divergence at 42 y for AD/MCI, 39 y for AD and 45 y for MCI. Afterwards, thalamus models diverge from control at 43 y for AD/MCI, 42 y for AD and 45 y for MCI. The divergence of amygdala models occurs at 44 y for AD/MCI, 41 y for AD and 49 y for MCI. Impact on global gray matter and white matter volume is observed later, with models diverging at 45 y and 48 y respectively for AD/MCI, at 46 y and 47 y respectively for AD and at 58 y and 54 y respectively for MCI. Finally, accumbens and caudate models diverge slightly later, but in a similar age range. Putamen and globus pallidus are the only deep gray matter structures for which models do not diverge from CN across the entire lifespan. Indeed, as assessed in Fig. 1, the confidence intervals of normal and pathological models always overlap.

**Figure 2:**
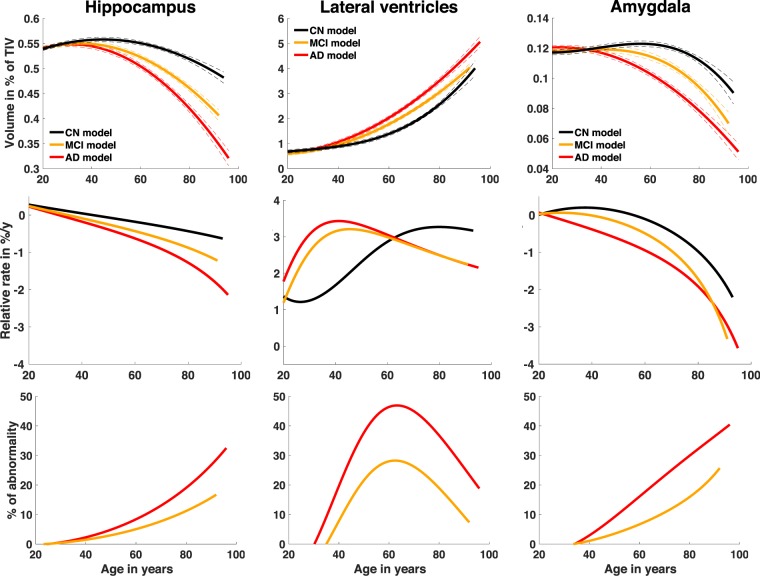
Hippocampus, lateral ventricles and amygdala models for CN, AD and MCI groups. The relative volumes (% total intracranial volume) are displayed according to the age in years across the entire lifespan. The prediction bounds are estimated with a confidence level at 95%. Relative rate of change is based on the first derivative of the model divided by the model and provided in % per year. Finally, percentage of abnormality (i.e. absolute relative difference compared to control) is estimated as the absolute difference between CN model and AD or MCI models divided by CN model. The model for CN group (N = 2944) is displayed in black, the model for MCI group (N = 2836) is displayed in yellow and the model for AD group (N = 2303) is displayed in red.

To further analyze well-known AD biomarkers, we propose a second analysis focusing on the hippocampus, the lateral ventricles and the amygdala. Figure 2 presents the lifespan models of these structures for CN, AD and MCI groups. Moreover, relative rate of change and abnormality percentages are provided.

First, divergence points of the models occur earlier for AD than for MCI (see Table 3 for exact time). As expected, models for MCI are in between AD and CN ones. Second, when using relative rate of change, amygdala and lateral ventricle exhibit a more pronounced relative rate of change compared to hippocampus. The maximum relative rate of changes for AD models of these structures are −3.6%/y for AG at 96 y, −2.1%/y for hippocampus at 96 y and 3.4%/y at 42 y for lateral ventricles. Contrary to hippocampus and amygdala, which show an increasing relative rate of change with age, lateral ventricles exhibits enlargement following an inverted U-shape. When considering abnormality percentage, an earlier abnormality increase is observed for hippocampus than for lateral ventricles and amygdala. This abnormality reaches a maximum of 32% for the AD model at 96 y. Abnormality appears later in life for lateral ventricles and amygdala and follows very different patterns for both. The lateral ventricles abnormality follows an inverted U-shape with a maximum of 47% at 63 y for the AD model. The amygdala abnormality has similar trend to that of the hippocampus abnormality. Amygdala volume reaches 40% of abnormality at 96 y for the AD model. Therefore, while hippocampus abnormality starts first, amygdala presents a greater abnormality at advanced age. Moreover, the abnormality observed in lateral ventricles is also important but its maximum is reached at 65 y. Afterwards, percentage of abnormality of lateral ventricles decreases to end at 19% at 96 y for the AD model. Therefore, at late age, the lateral ventricles show lower abnormalities than those of the hippocampus and the amygdala at the same ages.

**Figure 3:**
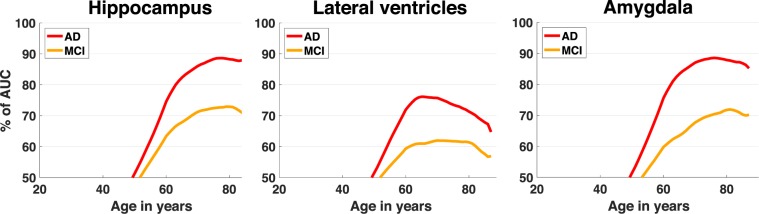
Classification accuracy across lifespan for hippocampus, lateral ventricles and amygdala volumes in term of AUC %. For each age, a classification is performed with LDA using all the samples in an interval of 10 y (i.e.,+/− 5 years). The AUC is estimated through a cross-validation procedure based on a repeated K-fold using 10 iterations and 10-fold. The average AUC obtained over the 10 repetitions is reported for all ages.

Finally, we propose a third analysis to estimate classification performance of the studied AD biomarkers according to the period of life. In the past, a large number of studies has been dedicated to AD patient classification for computer-aided diagnosis purpose^5,40–45^. In such studies, the classification performance of different biomarkers/features is compared to estimate their capability to distinguish patients from control subjects^46–48^. However, the evolution of the classification performance according to the period of life has not been studied. Therefore, we propose to estimate classification accuracy of hippocampus, lateral ventricles and amygdala volumes across the lifespan. Figure 3 presents the evolution of AUC across lifespan.

First, we can notice that the evolution of classification performance based on hippocampus and amygdala volumes are similar for the AD population with a plateau around 89% of AUC starting at 72 y. In contrast, for the MCI population, AUC trajectory of both regions does not behave similarly. Indeed, AUC increases faster and earlier for hippocampus volume than for amygdala volume. However, at the end, AUC trajectory for both regions converge to similar maximum around 73% at 80 y for hippocampus and around 72% at 80 y for amygdala. These AUC values are in line with literature dedicated to automatic classification of patients^40,45^. Finally, AUC based on lateral ventricles follows an inverted U-shape for AD population with a maximum of 76% around 65 y. For the MCI population, AUC shows a plateau around 60% from 60 y to 80 y.

## Discussion

In this paper, we investigate lifespan changes of the human brain in Alzheimer’s disease. The main novelty of this work is to propose pathological trajectory of brain structure over the entire lifespan. Consequently, it is possible to estimate when our normal and pathological models diverge. Moreover, our models enable to follow the dynamic of biomarker abnormality over the entire evolution of the pathology.

Our lifespan analysis using inferred models of brain trajectory in AD indicates that the hippocampus is the brain structure that exhibits the earliest divergence between cognitively normal model and pathological model. This model divergence is detectable early in life, at 39 y for the AD/MCI and at 37 y for the AD. The hippocampus model shares similar trends with another temporal lobe region, the amygdala, which presents model divergence at 44 y for AD/MCI and at 40 y for AD. It is noticeable that amygdala model is undergoing larger changes proportionally to its size compared to hippocampus. Finally, the lateral ventricles model presents an early divergence at 42 y for AD/MCI and at 39 y for AD. However, lateral ventricles enlargement occurring during normal aging reduces the abnormality of this structure after 60 y. Finally, the thalamus model shows early divergence at 43 y for AD/MCI and at 42 y for AD.

Our results presenting the hippocampus model as the first brain region diverging from normal aging model is in accordance with previous morphometric studies focused on the prodromal phase of the disease^12–15,49,50^. It is also in accordance with histopathological studies showing the temporal lobe as the starting point of the neurodegenerative process in AD^51^. In long follow-up studies, authors observed that incident cases of AD present morphometric difference in the hippocampus at least 10 years before the diagnosis^13,14,21^. Hippocampal atrophy has also been reported in several different transgenic mouse models of AD^52^, such as the amyloid precursor protein/presenilin2/Tau model, in which the volume reduction progresses with the pathology^53^, and it is associated with an enlargement of the lateral ventricles. *In vivo* MRI-measure of grey matter atrophy in AD has been described as a surrogate of the amount of neurofibrillary tangle revealed by post-mortem immunohistochemistry in human^54^. As this observation has been done in aging AD-diagnosed subjects, we could not ascertain that the mid-life atrophy revealed by our model is the result of the same Tau-pathophysiological process. Nevertheless, some recent studies revealed that temporal grey matter atrophy is associated to temporal Tau accumulation^55,56^ in clinically normal subjects. Moreover, a part of the effects of Tau accumulation on cognition is mediated by the temporal grey matter atrophy. On the other side, amyloid burden would be an independent aggravating factor of the process impacting temporal lobe regions, with an additive effect on cognition^57^. In fact, amyloid deposition in healthy elderly subjects, measured by the Pittsburgh compound B positron emission tomography, has been shown in a longitudinal study to be associated with later hippocampal atrophy and memory impairment^58^. All these results indicate that AD results from the cooccurrence of different pathological process, partially independent from each other and presenting different time course depending on the interplay between genetic and environmental factors. The time relationship between the different neuroimaging biomarkers is currently the subject of intensive research to describe early pathophysiological process associated to the disease. In addition to the atrophy and the Tauopathy, AD brain presents amyloid deposition. During the asymptomatic phase of the disease these biomarkers could be concomitantly or separately observed *in vivo*, leading to a proposed Amyloid/Tau/Neurodegeneration classification scheme to describe subjects during this silent phase^59^.

According to our results, the second structure of temporal lobe region diverging from the cognitively normal model is the amygdala, which is different from CN at 40 y for AD and at 44 y for MCI/AD. Atrophy of this structure has been repeatedly described in AD subjects, with a rate of change less important than^60,61^ or similar to^24^ the hippocampal one. In our model, we found that the time course of volume evolution of the amygdala is very closed to the one of the hippocampus. Importantly, after divergence, AD and CN models of the hippocampus and amygdala volumes never overlap across lifespan, in contrast to other deep gray matter structures models investigated in this study. This result highlights the specificity all along life of the medial temporal lobe alteration associated to the mnesic symptoms, which characterize the disease. The early reduction of amygdala volume has also been observed in the transgenic mouse model APPswe/PS1dE9 of AD, where neurodegeneration in the amygdala even precedes that found in the hippocampus^62^. The early divergence of the amygdala in the AD model is not surprising when considering the implication of emotion in memory. Indeed, the activity of basolateral and lateral nuclei of the amygdala is associated to a facilitation during the encoding phase and to an enhanced retrieval, these effects being mediated through the important interconnections between these structures and the hippocampus^63^. In addition, degradation of emotion processing ability is also observed in AD patients, as expected given the amygdala atrophy^64^. Moreover, the atrophy of the amygdala is likely contributing to the olfactory deficits associated with AD, since the cortical nuclei of the AG are associated with the processing of olfactory stimuli^65^. Hyposmia has been described in AD^66^, and olfactory deficits can substantially precede cognitive symptoms^67,68^. However, it has to be taken into account that pathological alterations in AD occur also in other olfactory structures^69,70^.

Based on our study, the volume of the lateral ventricles is also an early biomarker of AD, since its model diverges at 42 y for AD/MCI and at 39 y for AD. The potential of using lateral ventricles volume as AD biomarkers has been previously mentioned over restricted periods^27,71^. In this study, by analyzing the lifespan evolution of lateral ventricles abnormality, we showed that lateral ventricles abnormality decreases after 65 y. Therefore, the use of this biomarker is difficult for the late onset cases due to important lateral ventricles enlargement occurring during normal aging. However, it may be useful to discriminate cases around 65 y, an early age at which the AD diagnosis is particularly relevant because future potential treatment could be more effective in the early phases of the disease^72^. The importance of taking into account volume increase at advanced age in normal aging has been previously mentioned^26^. In the present study, early divergence of the models has been also observed for thalamus around 42 y. Thalamic atrophy was previously reported in AD literature^73,74^. However, we found that thalamus abnormality was very small (4.6% at 81 y). This may explain why only a small number of studies have mentioned that this structure could be affected by AD, because a large number of subjects are needed to detect such subtle atrophy.

In this study, to overcome the absence of longitudinal datasets with several decades of follow-up, we processed a massive number of cross-sectional MRI to generate a model of the volume trajectory in several brain structures in AD across the entire lifespan. We acknowledge that the use of cross-sectional data to analyze a dynamic process is not optimal. However, previous studies demonstrated that cross-sectional and longitudinal approaches produce similar age-related patterns in normal aging^75^ and similar models in AD^76^. Compared to previous longitudinal investigations, our results based on cross-sectional models are consistent with most previous findings on the importance and timing of hippocampus, amygdala and lateral ventricles alterations^27,77,78^. Although different in nature, it can be interesting to compare relative rates of change obtained with our models and annual rates of atrophy estimated on longitudinal data. The obtained values for relative rates of change are at the lower bound of expected range of annual atrophy rate reported in previous longitudinal literature^77,79,80^. However, our relative rates of change fits very well with the longitudinal rate of atrophy recently estimated with a multi-atlas method known to provide a less biased estimation^81^.

With respect to the estimated point of divergence between the CN and AD models, there is no longitudinal or cross-sectional MRI-based study literature over the lifetime with which to compare. However, our estimated point of divergence between models can be put into perspective with the detection of first AD signs in long follow-up longitudinal studies. First, prospective and longitudinal studies dedicated to autosomal dominant AD detected hippocampal atrophy up to 15 years before symptom onset^10^. Moreover, long follow-up population-based studies tracking cognition estimated that declines start from few years up to several decades before AD diagnosis^82–85^. Therefore, these results seem to confirm the presence of a long-lasting period of silence in AD, as discussed in^86^. Our models indicate that the age of 40 y is a critical period in the onset of the temporal lobe alteration. Midlife lifestyle factors such as diet^20^, physical activity^87^ and exposure to risk factors^88^ are associated with the risk of developing late life dementia; the life style factors of this critical period may specifically impact hippocampus leading to an increase vulnerability to dementia^19^. Consequently, exposure to risk factors (such as diabetes and smoking) occurring at this lifetime period should be considered in future studies to evaluate their implication in the early atrophy process.

We also proposed a classification analysis in order to highlight the importance of considering lifespan trajectory when evaluating biomarker performance. While most of the studies dedicated to automatic patient classification present only a global accuracy, our experience shows that biomarker efficiency changes according to the period of life. Therefore, computer-aided diagnosis study should test the accuracy of the biomarker at different time points.

Finally, our pathological model present three main limitations. First, we used normal control for the period [0–55] years. However, there is no alternative to simulate the AD model before 55 years. Second, by mixing AD and MCI subjects in the same model, we included false positive subjects in the pathological model since we do know that only a part of the MCI subjects will develop AD. However, we have also analyzed the AD and MCI models separately for the most relevant structures. Finally, in our model we consider that brain alteration caused by AD is a progressive and smooth phenomenon. The transition from CN to AD over the lifespan is viewed as a continuum. Such approach is not well-suited if brain modifications occurs suddenly. However, evidences such as slow accumulation of Amyloid-β^3,22,23^ or smooth and progressive atrophy of brain^3,10^ seem to assess that brain alterations occurs smoothly and progressively in AD.

## Conclusion

In this work, to analyze when AD brain model diverges from the cognitively normal model we build extrapolated lifespan models of AD brain structures by combining multiple large-scale databases. We found an early divergence of AD model from control model for hippocampus before 40 y followed by lateral ventricles and amygdala around 40 y. Moreover, we observed a similar abnormality evolution for the hippocampus and the amygdala. Finally, the model of ventricular enlargement shows that the volume of lateral ventricles reaches a peak of abnormality at 65 y before decreasing due to important enlargement in normal aging.
